# Measuring the Healthiness of the Packaged Food Supply in Australia

**DOI:** 10.3390/nu10060702

**Published:** 2018-05-31

**Authors:** Michelle Crino, Gary Sacks, Elizabeth Dunford, Kathy Trieu, Jacqui Webster, Stefanie Vandevijvere, Boyd Swinburn, Jason Y. Wu, Bruce Neal

**Affiliations:** 1 The George Institute for Global Health, University of New South Wales, Sydney, NSW 2042, Australia; edunford@georgeinstitute.org.au (E.D.); ktrieu@georgeinstitute.org.au (K.T.); jwebster@georgeinstitute.org.au (J.W.); jwu1@georgeinstitute.org.au (J.Y.W.); bneal@georgeinstitute.org.au (B.N.); 2 School of Public Health, Faculty of Medicine, The University of Sydney, Sydney, NSW 2042, Australia; 3 Centre for Population Health Research, Deakin University, Melbourne Burwood Campus, 221 Burwood Highway, Burwood, VIC 3125, Australia; gary.sacks@deakin.edu.au; 4 Carolina Population Center, The University of Chapel Hill, Chapel Hill, NC 27599, USA; 5 School of Population Health, University of Auckland, Auckland 1142, New Zealand; s.vandevijvere@auckland.ac.nz (S.V.); boyd.swinburn@auckland.ac.nz (B.S.); 6 Department of Epidemiology and Biostatistics, Imperial College London, London SW7 2AZ, UK

**Keywords:** nutrition, food supply, packaged foods, nutrient profiling, health star rating, INFORMAS, core foods, processed foods, dietary guidelines

## Abstract

The increasing availability of packaged foods plays a key role in nutritional transition. This study examined the healthiness of the Australian packaged food supply using a range of different metrics; 40,664 packaged products from The George Institute’s FoodSwitch database were included. Median and interquartile range (IQR) were determined for each measure of nutrient composition; mean and standard deviation (SD) for the measure based upon Health Star Rating (HSR); and proportions (%) for the measures based upon products with a higher HSR, classification of foods as either core or discretionary, extent of processing and proportions of foods that met reformulation targets for sodium, saturated fat and total sugars. Overall median (IQR) values were 1093 (1256) kJ/100 g for energy, 1.7 (6.3) g/100 g for saturated fat, 5.3 (21.4) g/100 g for total sugars, 163 (423) g/100 g for sodium and 50 (100) g or mL for serving size. Overall mean (SD) HSR was 2.8 (1.4), proportion with HSR < 3.5 was 61.8%, proportion of foods defined as discretionary was 53.0% and proportion of foods defined as highly processed was 60.5%. There were sodium targets set for 21,382/40,664 (53%) foods and achieved for 14,126/40,664 (35%). Corresponding figures for saturated fat were 328/40,664 (0.8%) and 130/40,664 (0.3%). Nutrient profiling, dietary guidelines and the extent of food processing provided comparable assessments of the nutritional quality of Australia’s packaged food supply. Individual measures of nutrient composition did not, but may be of value for identifying specific foods of concern.

## 1. Introduction

Packaged food now predominates food purchases in most countries [1,2], and the consumption of packaged foods in developing countries is rapidly increasing [3,4]. Technological developments in food processing along with advancements in food distributions systems have seen traditional wet markets for fresh foods replaced with supermarkets and hypermarkets which are abundant in packaged foods [5,6]. Usually, packaged foods are manufactured or processed before reaching the consumer and are more likely to have salt, sugar and fat added [5,6,7,8]. The increasing availability of packaged foods plays a key role in the nutrition transition, whereby nations move away from a traditional diet based on wholefoods to a diet of convenience [3,9,10]. Packaged food sales grew by 3% in Australia in 2016, with 58.8% of food and beverage products sold from retail and foodservice outlets being packaged foods [11,12]. 

The over-consumption of energy-dense, nutrient poor foods is a well-established risk factor for obesity and diet-related non-communicable diseases (NCDs) [4,5,13,14]. In 2013, the World Health Organization (WHO) developed a global NCD monitoring framework that included food environment-related risk factors such as the sale of unhealthy packaged foods high in salt, saturated fat and *trans* fat [15,16]. The framework identified approaches to monitoring national system responses to address these risks [15,16], but data on the healthiness of the food supply at the country-level are few [17]. Household expenditure surveys and national nutrition surveys, the most common measures of national dietary behaviours, capture important information but they do not provide adequate data to characterise and monitor the impact of actions of government and the food industry affecting the healthiness of the food supply [17,18]. 

INFORMAS (International Network for Food and Obesity/NCD Research, Monitoring and Action Support)—a global network of public-interest organisations and researchers—developed a framework to monitor, benchmark and support public and private sector actions to create healthy food environments [19]. Specific to food composition, the monitoring approach identified was to systematically collect information about nutrient composition of the food supply and to assess the energy density, salt (sodium), saturated fat, sugar, trans fat and portion sizes of packaged foods and beverages as well as their summary nutrient profile [17,20].

The objective of this research was to use INFORMAS measures to examine the nutrient composition of the Australian packaged food supply and explore additional measures based on dietary guidelines, extent of food processing and progress towards established reformulation targets. 

## 2. Methods

This was a cross-sectional examination of packaged foods available in supermarkets in Australia between August 2016 and August 2017. Assessment measures proposed by INFORMAS [17] were used and supplemented by methods based upon dietary guidelines, extent of processing and nutrient reformulation targets (Table 1). Specifically, we applied the Australian Health Star Rating (HSR) methodology based on nutrient profiling [21], the classification of foods as core or discretionary according to the Australian Dietary Guidelines (ADGs) [22,23], the classification of foods based upon their level of processing [24] and the proportion of foods that meet established reformulation targets for sodium, saturated fat and sugars based upon national or international recommendations (Table 1).

### 2.1. Data Source

Packaged food data (nutrient information, serving size and food category) were obtained from the 2016/17 Australian FoodSwitch database [25,26]. Briefly, the collection and processing of packaged food data from four large supermarkets follows standard processes [27] and is supplemented with crowd-sourced information for additional products [25,26]. Packaged food product data were only included in the analysis if nutrient information was available in either per 100 g or per 100 mL format. 

### 2.2. Food Categorisation

Products in the FoodSwitch database are assigned to one of 1271 food categories that group through a tree structure into 17 major food categories [25]. The major foods categories ‘Foods for specific dietary use’, ‘Vitamins and minerals’ and ‘Alcoholic beverages’ were excluded, leaving 14 major food categories for evaluation. Analysis of first order subcategories of the 14 major categories ‘Dairy’ and ‘Non-alcoholic beverages’ was also undertaken to illustrate specific points. The ‘Herbs and spices’ and ‘Variety packs’ first order subcategories were excluded, leaving 55 for evaluation.

### 2.3. Measures of Nutritional Quality

*Nutrient composition*—Selected risk-associated nutrients known to have adverse effects on an individual’s health (energy density, saturated fat, total sugars, sodium), as well as declared serving size were assessed. *Trans* fat and added sugars were excluded because labelling is not required under Australian law and most food products did provide this information. Targets for nutrient composition were identified [28] and nutrient levels in Australian packaged foods were compared against applicable Australian Government Food and Health Dialogue [29,30], New Zealand Heart Foundation [31] and United Kingdom [32,33] food reformulation targets. Where there were multiple reformulation targets for the same food category, the most recent target was used. If both average and maximum targets were available, the maximum target was used.

*Nutrient profiling summary score*—The nutrient profiling method used for this analysis was that underlying the Australian Health Star Rating (HSR) front of pack labelling system, a government-endorsed nutrient profiling method. The HSR system assigns packaged food products a rating between 0.5 (least healthy) and five stars (healthiest) in ten half-star increments based on the nutritional composition of the product [20,34]. The Australian FoodSwitch database captures the HSR when labelled on pack, and for instances when HSR is not available on pack, calculates this using energy, protein, saturated fat, total sugar and sodium values from product labelling. The HSR algorithm also requires information that is not always available on pack (fruit, vegetable, nut and legume (FVNL), concentrated FVNL and fibre). Where required nutrition data were absent, proxy values were estimated using information drawn from the back-of-pack ingredients list, generic food composition databases, or similar products using methods described previously [26]. We classified a product as ‘unhealthy’ if the product had a HSR < 3.5. This cut-off point is based on previous research indicating that healthy core foods with a HSR of ≥3.5 can be confidently promoted in public settings [35,36,37].

*Dietary* Guidelines—Foods were classified as core or discretionary based on definitions within the Australian Dietary Guidelines [22,23]. Core foods are foods that form the basis of a healthy diet (e.g., fruit, vegetables, lean meats, milk, yoghurts, cheeses, grains). Discretionary foods are energy-dense and nutrient-poor foods not necessary for providing the nutrients the body needs (e.g., sweetened soft drinks/cordials/waters, biscuits, chocolate, meat pies, butter, salty snacks). The relevant assignment was applied to each of the 1271 food subcategories.

*Extent of* processing—Foods were classified as ‘less processed’, ‘moderately processed’ or ‘highly processed’ using an adapted version of the NOVA classification framework [24]. This classification framework was used as previous research indicated it was easiest to apply [38]. The relevant assignment was applied to each of the 1271 food sub-categories and primary reporting was of the proportion ‘highly’ processed.

### 2.4. Analysis

Median and interquartile range (IQR) was determined for each measure of nutrient composition; mean and standard deviation (SD) for the measure of HSR; and proportions (%) for the measures based upon HSR < 3.5, classification of foods as either core or discretionary, extent of processing, and proportions of foods meeting established reformulation targets. Estimates were made overall, for each major food category and for illustrative sub-categories.

Coherence of the different measures of nutritional quality was assessed by ranking each of the 14 major food categories by each measure—lower energy density, saturated fat, total sugar, sodium, serving size, proportion HSR < 3.5, proportion discretionary and proportion highly processed, and higher mean HSR, being better. To enable a visual comparison, the upper five ranks were coloured light grey, the lower five dark grey and the middle four grey, with the data plotted as a heat map. Major food categories on the heat map were ordered for listing according to average rank across proportion HSR < 3.5, proportion discretionary and proportion highly processed. Quantitative analyses were done using Minitab™ 17 Statistical Software (Minitab Inc., State College, PA, USA).

## 3. Results

Nutrient composition and proportion of products meeting a reformulation target were assessed for 40,664 (100%) foods; nutrient profile for 38,451 (95%) foods; core/discretionary status according to Australian Dietary Guidelines for 37,174 (91%) foods; and extent of processing for 37,348 (92%) foods.

### 3.1. Measures of Healthiness in Major Food Categories

**Figure 1:**
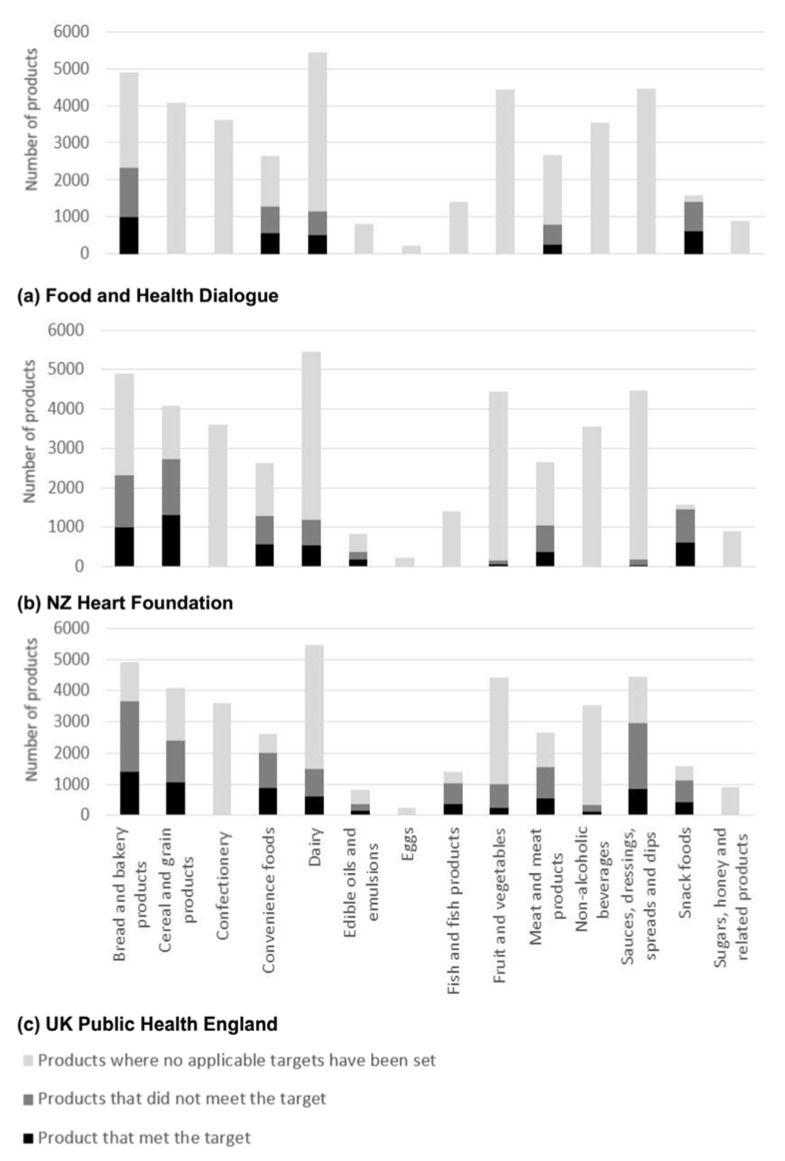
Number of Australian packaged food products without, with and meeting the sodium reformulation target set by either (**a**) Food and Health Dialogue [28]; (**b**) New Zealand Heart Foundation [31]; and/or (**c**) United Kingdom (UK) Public Health England *. * Where averages and maximum limits we both specified, maximum limits were used for analysis.

**Figure 2:**
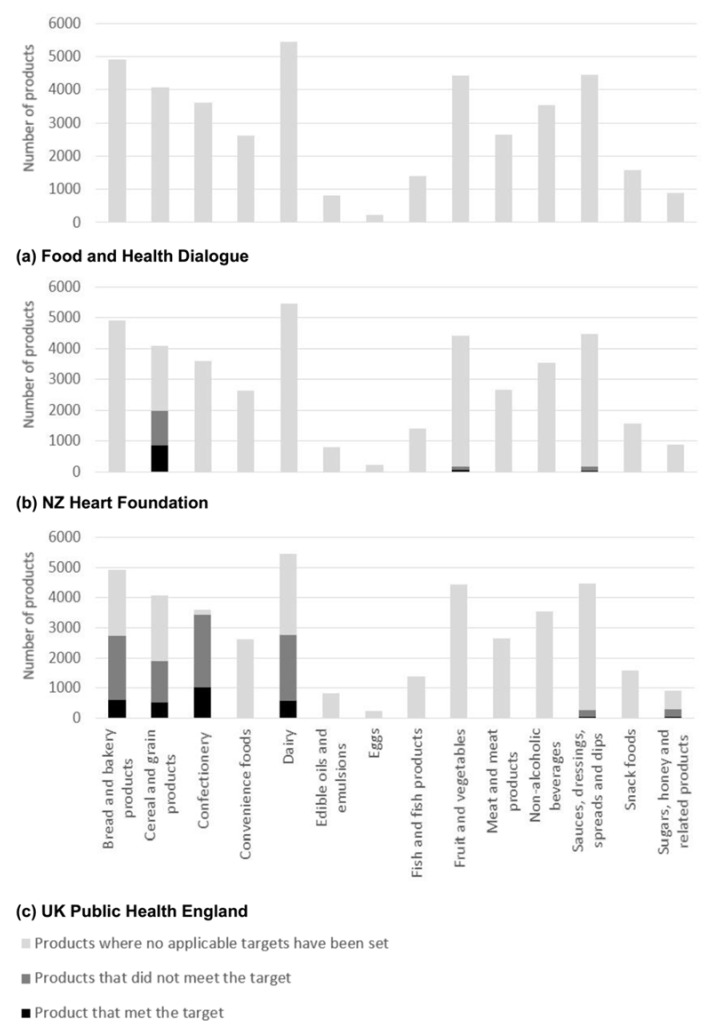
Number of Australian packaged food products without, with and meeting total sugars reformulation target set by either (**a**) Food and Health Dialogue [28]; (**b**) New Zealand Heart Foundation [31]; and/or (**c**) United Kingdom (UK) Public Health England *. * Where averages and maximum limits we both specified, maximum limits were used for analysis.

**Figure 3:**
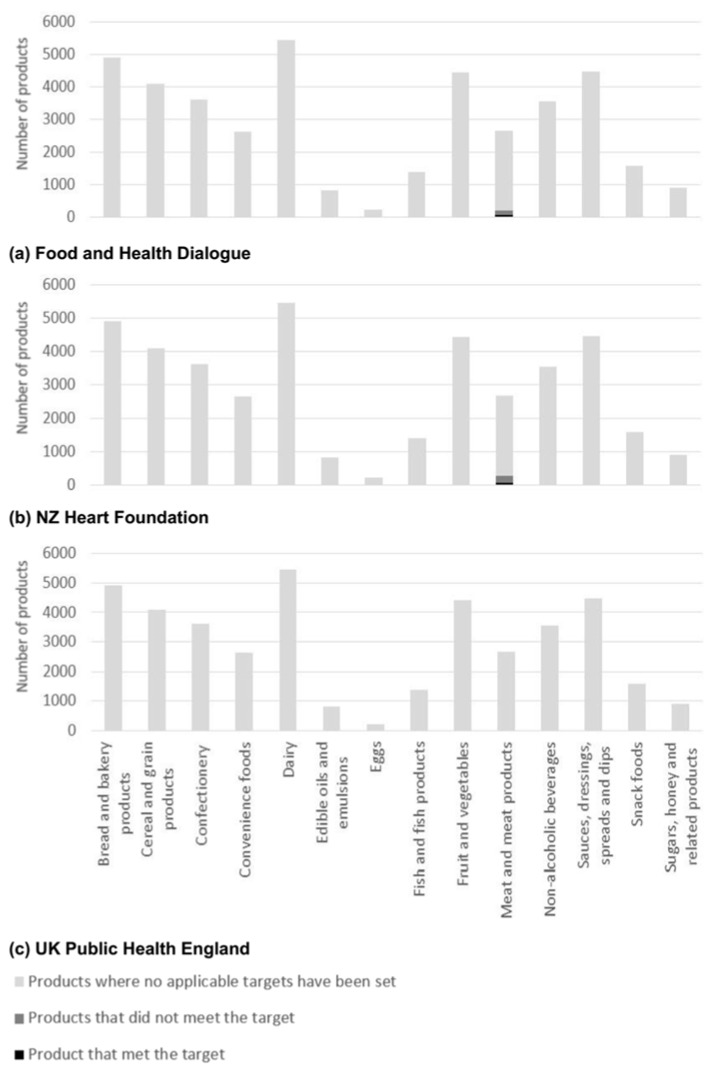
Number of Australian packaged food products without, with and meeting saturated fat reformulation target set by either (**a**) Food and Health Dialogue [28]; (**b**) New Zealand Heart Foundation [31]; and/or (**c**) United Kingdom (UK) Public Health England *. * Where averages and maximum limits we both specified, maximum limits were used for analysis.

*Nutrient composition and reformulation targets*—Median and IQR values varied extensively between nutrients and across food categories (Table 2) and there was limited correlation of adverse levels of nutrients across food categories. For example, ‘Edible oils and emulsions’ had the highest energy density (3380 kJ/100 g) and ‘Non-alcoholic beverages’ the lowest (175 kJ/100 mL), though corresponding median serving sizes were 14 g and 250 mL respectively (Table 2). Sodium reformulation targets were defined by the FHD, NZ HeartSafe and UK (Figure 1) for 21,382 products; total sugar targets by NZ HeartSafe and the UK (Figure 2) for 9947 products and saturated fat targets by the FHD and NZ HeartSafe for 328 products (Figure 3). Salt targets were achieved for 14,126/40,664 (35%) products, sugar targets for 3754/40,664 (9%) and saturated fat targets for 130/40,664 (0.3%).

*Nutrient profiling summary score*—Overall, the Australian packaged food supply had a mean HSR of 2.8 (1.4) out of 5.0. HSR varied substantially across categories and within categories (Table 2). Four out of 14 categories had a mean HSR greater than 3.5: ‘Eggs’, ‘Fruit, vegetables, nuts and legumes’, ‘Seafood and seafood products’ and ‘Cereal and grain products’. Food categories with the lowest mean HSR were ‘Confectionery’ (1.3 (0.8)) and ‘Sugars, honey and related products’ (1.3 (0.9)). Approximately two thirds (60.8%) of Australian packaged products has a HSR < 3.5.

*Dietary guidelines*—There were 19,688 (53%) Australian packaged foods defined as discretionary and 17,486 (47.0%) defined as core (Table 2), with seven out of 14 categories comprising 50% or more discretionary foods. For ‘Confectionery’, ‘Snack foods’ and ‘Sugars, honey and related products’ all packaged foods were classified as discretionary whereas for ‘Eggs’ and ‘Seafood and seafood products’ all were classified as core.

*Extent of processing*—There were 22,602 (60.5%) highly processed, 6797 (18.2%) moderately processed and 7949 (21.3%) less processed foods (Table 2). The ‘Convenience food’ (97.8%) and ‘Confectionery’ (96.8%) categories had the highest proportion of highly-processed foods with ‘Eggs’ (0%) lowest, and ‘Fruit, vegetables, nuts and legumes’ (12.5%) next lowest.

### 3.2. Measures of Food Healthiness in Illustrative Food Sub-Categories

More detailed analysis of the ‘Dairy’ and ‘Non-alcoholic beverages’ subcategories highlighted the considerable variation in nutritional quality of the foods and beverages included within each major food category (Table 3). The ‘Non-alcoholic beverages’ category, for example, had energy density values that ranged from a median of 80 kJ/100 mL for the ‘Waters’ sub-category to 618 kJ/100 mL for ‘Beverage mixes’. Likewise, within ‘Dairy’ the median saturated fat varied from 23.0 g/100 g for ‘Cream’ down to 1.2 g/100 g for ‘Milk’ with equally large differences for the nutrient profiling measure (mean HSR: 1.4 (0.8) for ‘Cream’ versus 3.8 (1.0) for ‘Milk’). 

### 3.3. Comparison of Different Measures of Healthiness

**Figure 4:**
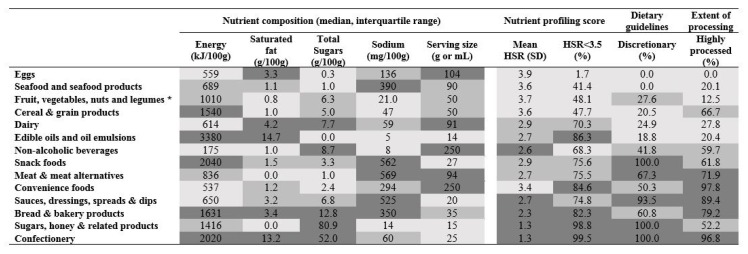
Coherence of ranking of the healthiness of 14 Australian packaged food categories according to nutrient composition, nutrient profile, dietary guidelines and extent of processing. The upper five ranked for each measure were coloured light grey, the lower five dark grey and the middle four medium-grey. Categories on the heat map were listed according to average rank across proportion HSR < 3.5, proportion discretionary and proportion highly processed. * Excludes herbs and spices. g = grams; mL = millilitres; mg = milligrams. Discretionary foods: energy-dense and nutrient-poor foods not necessary for providing the nutrients the body needs [22,23]. Highly-processed foods: multi-ingredient industrially formulated mixtures processed to the extent that they are no longer recognizable as their original plant/animal source and not typically consumed as additions [24].

There was broad coherence between the nutritional quality of different food categories defined according to the different summary measures employed (Figure 4). Median levels of energy, saturated fat, total sugars and sodium were mostly, though not always, aligned with assessments of nutritional quality based upon dietary guidelines and extent of processing (Table 4). Median saturated fat, for example, was higher in moderately processed foods (2.6 g/100 g) compared to highly-processed foods (1.8 g/100 g) primarily because large numbers of dairy products are defined as moderately processed. In the more granular analyses done by major food categories, however, rankings based upon nutrient compositions were inconsistent with each other and were not associated with rankings based upon nutrient profiling, dietary guidelines or extent of food processing (Figure 4). There was no clear pattern associating serving size with assessments based upon other measures. 

## 4. Discussion

Broadly consistent assessments of the nutritional quality of the Australian food supply were obtained using methods based upon nutrient profiling, dietary guidelines and extent of food processing. The observation that simpler methods based upon dietary guidelines and extent of food processing [7,39,40,41] provided comparable high-level findings to the more intensive nutrient profiling process is important because these methods may be more feasible in settings where detailed nutritional data and resources are limited [42,43]. Methods based upon dietary guidelines and extent of food processing are, however, unlikely to provide the detailed insight provided by nutrient profiling [38], which has a demonstrated capacity to discriminate between the healthiness of products within fine subcategories. For example, processed foods do not all have a poor nutrient composition with products suchas hummus being classified as highly-processed despite a substantially better nutrient composition than many other highly processed foods such as processed meats or sugar-sweetened beverages.

The assessments based upon individual nutrients or serving size, by contrast, did not provide assessments of nutritional quality that were coherent across food categories or nutrients. Neither were the rankings of the nutritional quality of the food categories based upon individual nutrients consistent with those obtained using nutrient profiling, dietary guidelines or extent of processing. The nutrient values did identify specific aspects of concern about food categories that might be targeted for action. 

Assessments based upon achievement of nutrient reformulation targets are currently of limited value because targets have been set for only the minority of foods, and the foods with targets set are unlikely to be representative sample of all foods. The proportion of foods with a target set, and progress against a full set of targets might, however, be another useful measure by which to hold government and the food industry accountable for progress towards public health nutrition initiatives [17,19,44]. Consistency of targets across jurisdictions would simplify policy development, the implementation of interventions, and program monitoring. If targets are applied at a fine sub-category level this is a highly plausible approach and while some limited tailoring to local context may be required, most problems with the packaged food supply are not bounded by geography, and there is a strong argument for greater standardisation and global coordination.

A key strength of these analyses is the large and diverse dataset upon which the assessments were made, and the ability to calculate and compare multiple different measures of nutritional quality. This study reports findings for more complex approaches such as nutrient profiling which quantitatively summarise multiple dimensions of nutritional quality, as well as simpler and more pragmatic methods such as dietary guidelines and extent of processing. In addition to the practicalities of data collection, it is also possible that different measures will be of value for the assessment of different aspects of food policies seeking to improve the nutritional quality of the food supply.

Important weaknesses are the restriction of the data to Australian products, which raises uncertainties about the broader generalisability of the findings to other settings. For every measure, weighting of estimates for sales volumes would enhance the insight provided [45] although crude estimates have been shown previously to capture key characteristics of the packaged food supply [37,46]. In the absence of sales data, an alternate approach would be to utilise data from national dietary surveys to examine the proportion of daily energy that comes from the main food categories. For example, according to the 2011–12 Australian Health Survey, 35% of an adult Australian’s energy intake comes from discretionary foods. The assessment methods reported here are an incomplete set of all possible methods but cover the main types of approaches possible. The World Health Organization’s has criteria that define eligibility for marketing food products to children [47] and the Food Standards Australia New Zealand has another set of Nutrient Profiling Score Criteria [2] that determine the eligibility for products to carry health and nutrient content claims, that might be studied in future. Similarly, there are tens of other nutrient profiling methods [48,49,50,51,52] and a range of different systems for defining the extent of food processing [24,38,53,54,55] that could also be evaluated.

Another important weakness is the absence of data on *trans* fat because *trans* fat is a strong determinant of disease risk that can be addressed by proven policy interventions [56,57]. Likewise, the reliance on data describing total sugar is sub-optimal because it is free sugars that are the primary health concern and the target of interventions. Legislation of added sugar labelling has been passed in the United States [58,59] and should be adopted in Australia. It should also be a part of the standard Codex recommendation [60] for nutrient declarations. This paper has reported only on packaged foods but many of the methods could be extended and applied to assess foods served in restaurants or other foodservice establishments. Application of the assessment process to encompass the entire food environment might provide for a more complete assessment and more holistic decision-making. 

Data of this type have multiple potential uses. The comparisons made against reformulation targets provide a direct assessment of government action and highlight areas for food industry action. Between country comparisons of the nutritional quality of foods in conjunction with data describing food environment features might also provide insight into the food system interventions most likely to be effective. More granular reporting of comparisons between individual products and major companies would be highly newsworthy, and has the potential to drive corporate product ranging and formulation decisions [17,61,62,63,64].

## 5. Conclusions

These analyses have highlighted the feasibility and utility of a series of different approaches to measuring the nutritional quality of the Australian food supply. All measures indicate significant opportunity for improving the healthiness of Australian foods. Sequential analyses could provide a detailed assessment of the changing nature of the packaged food supply in Australia, the actions required to enhance nutritional quality and the effectiveness of programs that are implemented. There is a strong case for expecting that comparable analyses in other countries would provide similar insights and opportunities.

## Figures and Tables

**Table 1:** Measures and associated rationale used to assess the healthiness of the Australian packaged food supply.

Measure	Rationale
Nutrient composition	There are well established associations of individual nutrients with negative and positive health outcomes [1,2,3,4]. Key nutrients of public health relevance are routinely reported on food packaging for Australia and many other countries.
Nutrient profiling summary scores	Nutrient profiling systems can be used to combine multiple dimensions of a food (positive and negative components) into a single continuous metric providing a relatively easy comparison across a range of different food types [5]. The Health Star Rating (HSR) front of pack labelling system is one such system that has been implemented in Australia [6,7], but a wide range of nutrient profiling systems are available [5,8,9,10]. Systems based upon fewer and widely reported nutrient values may have greater utility since full data availability aids the application of a nutrient profiling system.
Dietary guidelines	A high-level classification of foods that can typically be done without the need for detailed nutritional data [11,12]. The Australian Dietary Guidelines are a locally applicable example that classify foods as ‘core’ or ‘discretionary’, but definitions and approaches to categorisation of foods vary around the world.
Extent of processing	A high-level method that classifies foods on the basis of the degree of processing applied to the food that can typically be done without the need for detailed nutritional data [11,12,13,14,15,16,17,18,19]. For this analysis we categorised foods into one of three categories: less processed, moderately processed and highly processed foods based upon methodology proposed by Poti et al. [20].
Reformulation targets	Progress towards establishing of reformulation targets for risk-associated nutrients and/or the proportion of products that meet these targets may indicate the healthiness of a nation’s food supply. This assessment requires detailed nutritional information about individual products [21,22].

**Table 2:** Assessment of the nutritional quality of the Australian packaged food supply using multiple measures *.

	Number of Products	Nutrient Composition (Median (IQR))					Nutrient Profiling Summary Score		Dietary Guidelines	Extent of Processing
		Energy (kJ/100 g)	Saturated Fat (g/100 g)	Total Sugars (g/100 g)	Sodium (mg/100 g)	Serving Size (g or mL)	Mean HSR (SD)	HSR < 3.5 (%)	Proportion ‘Discretionary’ (%)	Proportion Highly Processed (%)
Bread & bakery products	4906	1631 (717)	3.4 (8.4)	12.8 (30.3)	350 (290)	35 (46)	2.3 (1.2)	82.3	60.8	79.2
Cereal & grain products	4076	1540 (254)	1.0 (2.8)	5.0 (18.5)	47 (250)	50 (65)	3.6 (1.0)	47.7	20.5	66.7
Confectionery	3606	2020 (682)	13.2 (17.7)	52.0 (17.0)	60 (73)	25 (9)	1.3 (0.8)	99.5	100.0	96.8
Convenience foods	2629	537 (464)	1.2 (2.2)	2.4 (2.3)	294 (174)	250 (170)	3.4 (0.6)	84.6	50.3	97.8
Dairy	5448	614 (933)	4.2 (13.3)	7.7 (12.8)	59 (194)	91 (140)	2.9 (1.3)	70.3	24.9	27.8
Edible oils and oil emulsions	812	3380 (682)	14.7 (18.1)	0.0 (1.0)	5 (340)	14 (5)	2.7 (1.3)	86.3	18.8	20.4
Eggs	231	559 (0)	3.3 (0.0)	0.3 (0.0)	136 (0)	104 (14)	3.9 (0.0)	1.7	0.0	0.0
Seafood and seafood products	1392	689 (380)	1.1 (1.4)	1.0 (1.7)	390 (236)	90 (35)	3.6 (0.8)	41.4	0.0	20.1
Fruit, vegetables, nuts and legumes †	4427	1010 (1785)	0.8 (4.0)	6.3 (29.9)	21.0 (235)	50 (75)	3.7 (1.0)	48.1	27.6	12.5
Meat and meat alternatives	2657	836 (454)	0.0 (0.1)	1.0 (1.1)	569 (582)	94 (75)	2.7 (1.3)	75.5	67.3	71.9
Non-alcoholic beverages	3543	175 (98)	1.0 (3.2)	8.7 (6.1)	8 (10)	250 (100)	2.6 (1.6)	68.3	41.8	59.7
Sauces, dressings, spreads & dips	4464	650 (845)	3.2 (7.8)	6.8 (16.5)	525 (725)	20 (40)	2.7 (1.3)	74.8	93.5	89.4
Snack foods	1578	2040 (360)	1.5 (3.5)	3.3 (4.7)	562 (369)	27 (15)	2.9 (1.2)	75.6	100.0	61.8
Sugars, honey & related products	895	1416 (340)	0.0 (1.0)	80.9 (30.1)	14 (22)	15 (20)	1.3 (0.9)	98.8	100.0	52.2
Total	40,664	1093 (1256)	1.7 (6.3)	5.3 (21.4)	163 (423)	50 (100)	2.8 (1.4)	70.8	53.0	60.5

* Data rounded to the nearest whole number for energy, sodium, and serving size, and to one decimal space for saturated fat, total sugars and proportion discretionary/highly-processed. † Excludes herbs and spices. IQR = interquartile range; SD = standard deviation; HSR = Health Star Rating; kJ = kilojoules; g = grams; mL = millilitres; mg = milligrams. Discretionary foods: energy-dense and nutrient-poor foods not necessary for providing the nutrients the body needs [22,23]. Highly-processed foods: multi-ingredient industrially formulated mixtures processed to the extent that they are no longer recognizable as their original plant/animal source and not typically consumed as additions [24].

**Table 3:** Sub-category nutritional assessment analysis for dairy and non-alcoholic beverages categories using multiple measures *.

	Number of Products	Nutrient Composition (Median (IQR))					Nutrient Profiling		Dietary Guidelines	Extent of Processing
		Energy (kJ/100 g)	Saturated Fat (g/100 g)	Total Sugars (g/100 g)	Sodium (mg/100 g)	Serving Size (g/mL)	Mean HSR (SD)	HSR < 3.5 (%)	Proportion ‘Discretionary’ (%)	Proportion Highly Processed (%)
**Dairy (total)**	5077	614 (933)	4.2 (13.3)	7.7 (12.8)	59 (194)	91 (140)	2.9 (1.3)	70.3	44.4	36.5
Cheese	1315	1440 (480)	17.8 (6.6)	1.0 (0.6)	647 (267)	25 (4)	2.8 (1.3)	70.3	0.0	15.7
Cream	168	1390 (622)	23.0 (12.3)	3.3 (1.5)	33 (28)	25 (30)	1.4 (0.8)	99.4	91.1	0.0
Desserts	339	577 (819)	2.2 (4.7)	14.7 (8.4)	67 (81)	100 (45)	2.6 (0.9)	98.5	72.3	100.0
Ice cream & edible ices	830	844 (653)	6.6 (9.7)	22.3 (6.2)	52 (33)	70 (29)	2.1 (0.8)	98.0	100.0	100.0
Milk	1184	266 (112)	1.2 (1.6)	5.0 (3.9)	44 (12)	250 (0)	3.8 (1.0)	38.4	3.1	3.1
Yoghurt & yoghurt drinks	1241	429 (204)	2.4 (2.9)	11.1 (6.8)	56 (19)	140 (70)	2.9 (1.4)	70.5	0.0	0.0
**Non-alcoholic beverages (total)**	3352	175 (98)	1.0 (3.2)	8.7 (6.1)	8 (10)	250 (100)	2.6 (1.6)	68.3	70.4	66.6
Beverage mixes	80	618 (1228)	0.1 (1.0)	13.5 (28.6)	25 (113)	120 (230)	1.3 (1.0)	93.7	100.0	100.0
Coffee & tea	716	264 (1586)	1.0 (2.4)	3.5 (46.2)	12 (55)	170 (185)	2.0 (1.2)	89.8	21.2	35.2
Cordials	206	102 (88)	0.0 (0.0)	6.0 (6.0)	6 (8)	250 (50)	1.6 (0.4)	100.0	100.0	0.0
Electrolyte drinks	74	104 (27)	0.0 (0.0)	5.9 (0.3)	30 (23)	600 (350)	1.6 (0.7)	97.3	100.0	100.0
Energy drinks	131	192 (174)	0.0 (0.0)	10.6 (9.8)	48 (43)	330 (250)	1.1 (0.5)	100.0	100.0	100.0
Fruit and vegetable juices	1221	180 (37)	0.0 (0.1)	9.3 (2.6)	5 (4)	250 (50)	4.2 (1.4)	19.9	0.0	55.3
Soft drinks	605	124 (175)	0.0 (0.0)	7.4 (10.5)	11 (8)	300 (125)	1.5 (0.4)	100.0	100.0	100.0
Waters	319	80 (98)	0.0 (0.0)	3.8 (5.4)	12 (16)	258 (200)	1.8 (0.5)	98.7	42.3	42.3

* Data rounded to the nearest whole number for energy, sodium, and serving size, and to one decimal space for saturated fat and total sugars. IQR = interquartile range; SD = standard deviation; HSR = Health Star Rating; kJ = kilojoules; g = grams; mL = millilitres; mg = milligrams.

**Table 4:** Assessment of nutritional quality of the Australian packaged food supply by ‘core’ and ‘discretionary’ classification and extent of food processing *.

	Number of Products	Nutrient Composition (Median (IQR))					Nutrient Profiling Summary Score	
		Energy (kJ/100 g)	Saturated Fat (g/100 g)	Total Sugars (g/100 g)	Sodium (mg/100 g)	Serving Size (g or mL)	Mean HSR (SD)	HSR < 3.5 (%)
**Dietary guidelines**								
Core foods	17,486	844 (1218)	1.0 (3.5)	3.4 (7.6)	90 (379)	90 (140)	3.6 (1.1)	50.1
Discretionary foods	19,688	1330 (1250)	2.6 (8.6)	14.8 (39.3)	224 (484)	30 (62)	2.1 (1.2)	90.4
**Extent of processing (three levels)**								
Less processed foods	7949	642 (1261)	1.0 (3.2)	3.0 (6.4)	34 (172)	75 (95)	3.5 (1.2)	47.8
Moderately processed foods	6797	1166 (1221)	2.6 (7.7)	5.6 (11.6)	184 (525)	44 (105)	3.0 (1.3)	64.2
Highly processed foods	22,602	1180 (1173)	1.8 (7.0)	8.0 (28.5)	250 (440)	50 (100)	2.5 (1.3)	81.0
**Extent of processing (two levels)**								
Not highly processed †	14,747	880 (1335)	1.6 (5.1)	3.9 (9.4)	55 (375)	50 (100)	3.2 (1.3)	55.4
Highly processed foods	22,602	1180 (1173)	1.8 (7.0)	8.0 (28.5)	250 (440)	50 (100)	2.5 (1.3)	81.0

* Data rounded to the nearest whole number for energy, sodium, and serving size, and to one decimal space for saturated fat and total sugars. † Products not classified as ‘not highly processed’ are a combination of low and moderately processed foods. IQR = interquartile range; SD = standard deviation; HSR = Health Star Rating; kJ = kilojoules; g = grams; mL = millilitres; mg = milligrams. Core foods: foods are foods that form the basis of a healthy diet [22,23]. Discretionary foods: energy-dense and nutrient-poor foods not necessary for providing the nutrients the body needs [22,23]. Less-processed foods: includes unprocessed/minimally processed foods, processed basic ingredients and foods processed for basic preservation [24]. Moderately processed foods: single minimally or moderately processed foods with addition of flavour additives for the purpose of enhancing flavour. Also includes grain products made from whole-grain flour with water, salt, and/or yeast [24]. Highly-processed foods: multi-ingredient industrially formulated mixtures processed to the extent that they are no longer recognizable as their original plant/animal source and not typically consumed as additions [24].
